# Association of adult attachment with delays in accessing specialist care in women with ovarian cancer

**DOI:** 10.1080/07347332.2022.2025510

**Published:** 2022-02-03

**Authors:** Soumitra Shankar Datta, Lindsay Fraser, Matthew Burnell, Shazia Nasreen, Manisha Ghosh, Aparupa Ojha, Tania Saha, Asima Mukhopadhyay, Anne Lanceley, Usha Menon

**Affiliations:** a Department of Palliative Care and Psycho-oncology, Tata Medical Centre, Kolkata, India; b MRC Clinical Trials Unit, Institute of Clinical Trials & Methodology, University College London, London, UK; c Department of Gynaecological Oncology, Tata Medical Centre, Kolkata, India; d UCL EGA Institute for Women’s Health, University College London, London, UK

**Keywords:** adult attachment, delay, interval to diagnosis, LMIC, oncology, ovarian cancer

## Abstract

**Objective:**

Advanced stage at diagnosis and delayed presentation are common in ovarian cancer (OC). The objective of the current study was to explore the association of adult attachment pattern with delays in accessing specialist oncology care in patients with OC.

**Methods:**

A cross-sectional structured interview study of patients with OC presenting to an Indian cancer center was undertaken. Consenting patients completed Experiences of Close Relationships–Relationship Style questionnaire (ECR-RS) and Medical Outcome Survey–Social Support Survey (MOS–SSS). Multivariate linear regression with “time to presentation to cancer specialist” as the dependent variable was undertaken.

**Results:**

In all, 132 of 155 (85%) patients with OC who were invited were interviewed. An increased ECR-RS attachment anxiety score (*P* = .01) and being part of a multigenerational extended household (*P* = .04) were both independently associated with delay in presentation to a cancer specialist. There was no association between delay in presentation and social support.

**Conclusions:**

Among patients with OC, adult attachment may contribute to delays in presentation. It may be important for the cancer symptom awareness efforts in primary care to include educating physicians on recognizing and interacting with patients with insecure attachment styles. The association of delays in presentation for women with OC living in multigenerational extended households needs more indepth exploration.

## Background

Ovarian cancer (OC) continues to be a disease with high case fatality ratio. In 2020, 313,959 women worldwide were diagnosed and 207,252 died of the disease.^1^ A key factor responsible for the high mortality is diagnosis at a late stage.^2,3^ This has been attributed to delays in presentation by patients as well as delays in onward referral to cancer specialists by primary care physicians.^4^ Delays in initiating treatment are associated with poorer quality of life, increased severity of physical symptoms, and reduced overall survival in women with OC.^5^ This has led to ongoing international efforts^6^ to better understand and address these delays and optimize referral pathways by both mapping time intervals to diagnosis and treatment^7,8^ and exploring causes and interventions that may facilitate earlier presentation to a cancer specialist.^9^ However, there has been limited focus on psychological risk factors that might contribute to these delays in presentation in women with OC.

Recent research has shown that adult attachment can influence help-seeking behavior.^10^ Adult attachment is the cluster of attitudes and behavioral and emotional expressions in close relationships that are particularly prominent when an individual is under threat.^11^ In the model proposed by Bartholomew and Horowitz,^12^ each individual has a personal attachment pattern that can be mapped by the two dimensions of attachment anxiety and attachment avoidance.^10,12,13^ Attachment anxiety is the degree of discomfort one feels due to separation from attachment figures.^11^ Attachment avoidance is the extent to which one is distressed by crowding or closeness to the attachment figure.^11^ It has been studied across multiple countries including India and found to have similar dimensions. Individuals may have predictable responses to serious stressors like a cancer diagnosis or a traumatic life event.

There is growing evidence that high attachment anxiety and attachment avoidance may be related to suboptimal health behavior.^14^ In particular, a study of Danish patients with cancer has reported that high attachment anxiety was associated with increased delay in seeking help from general practitioners.^15^ A number of potential mechanisms have been proposed to explain this finding. The first is related to the association between attachment and the capacity for narrative coherence.^16^ Individuals with high attachment anxiety are less able to coherently narrate their history. Their stories are often characterized by intense affect, overwhelming details, lack of clarity, digression, circumstantiality, and lack of context. Their poorly articulated distress and the tension and anxiety often lead to the listener (including health workers) withdrawing from the encounter, even prior to collecting the information that is needed to offer appropriate advice and support.^16,17^ In addition, individuals with high attachment anxiety may endure difficulties in isolation without recruiting support as they perceive that required help will not be forthcoming. Finally, patients who score high on attachment avoidance may be skeptical of seeking help from others, be it friends, family members, or professionals.^18,19^ All of these could potentially lead to treatment delays.

We undertook a study to explore whether the adult attachment of patients with OC is associated with delays in accessing specialist cancer care. Our specific hypotheses were that (1) patients with OC with high attachment anxiety are more likely to experience longer time intervals in presenting to cancer specialists than patients with low attachment anxiety and (2) those with high attachment avoidance are more likely to experience longer time intervals in presenting to cancer specialists than patients with low attachment avoidance.

## Methods

The study used a cross-sectional design of patients with incident OC at a state-of-the-art cancer center in India. The objective was to explore the association between adult attachment anxiety and attachment avoidance and the time interval to presentation to cancer specialists in women with OC. The study was approved by the institutional ethical review board (IRB 2015/005). Given that this study is exploratory in nature, we did not perform a formal power analysis calculation.

### Participants

All women attending the hospital during the study period who were diagnosed with OC and who were aged 18 years and older were invited to participate. There was no upper age limit. The exclusion criteria were patients (1) too ill to be interviewed as per their clinician, (2) unable to provide informed consent, or (3) unable to converse in Bengali (local language) or English. Eligible patients were approached by their clinician when they attended the outpatient facility prior to start of treatment (surgery or chemotherapy). Women who consented were interviewed by a trained clinical psychologist in a private room away from the busy clinic. The interviewer was not part of the patient’s oncology team and was unaware of stage or other details of the disease.

### Measures

The following research tools/questionnaires were administered to collect baseline and outcome information:

#### Sociodemographic questionnaire

Age, urban/rural residence, educational attainment, marital status, number of family members, and type of family unit (nuclear versus multigenerational extended family) in childhood and at present. Multigenerational extended family is a family that includes in one household near relatives across the generations such as grandparents, parents, and male siblings and their spouses and their children.

#### Disease and treatment questionnaire

Date of onset and description of initial symptoms, the first health professional consulted, and the date they first presented to a cancer specialist. The dates for the first consultation with the cancer specialist were verified from the hospital records after the participant interviews. The cancer specialists included could be medical, clinical, gynecological, or surgical oncologists. Details of histology and stage were retrieved from medical records at a later date.

Adult attachment was assessed using the Experiences in Close Relationships–Relationship Structure questionnaire (ECR-RS).^13^ It uses a 7-point rating scale ranging from “strongly disagree” to “strongly agree” to measure two dimensions of attachment—avoidance and anxious—on 9 statements of the scale. Example statements that explore attachment anxiety include “It helps to turn to people in times of need” and “I talk things over with people.” Example statements of attachment avoidance include “I often worry that other people do not care for me” and “I’m afraid that other people may abandon me.” Attachment avoidance is calculated by averaging the score of items 1 through 6, of which items 1 through 4 are reverse-coded. Attachment anxiety is scored by averaging items 7 through 9. The ECR-RS was validated in a global study of 21,000 individuals that included India. The reported Cronbach’s alpha for the ECR-RS anxiety scores was 0.85 and for the ECR-RS avoidance scores was 0.88.^13^ We chose this instrument as recent research has shown that a two-dimensional model of adult attachment is better suited for conceptualizing and measuring individual differences in attachment.^20^ The ECR-RS was translated to Bengali by bilingual professionals who were not part of the study team following accepted guidelines.^21^ An expert group consisting of psychiatrists and clinical psychologists reviewed the translated ECR-RS for conceptual equivalence. The Bengali ECR-RS was back-translated into English to ensure that the translated instrument reflected same item content as the original version. It was then piloted in a group of 10 patients and found to be satisfactory. The expert group proofread the final translated version of the ECR-RS.

Social support was assessed by the Medical Outcome Survey–Social Support Survey (MOS–SSS).^22^ This is a 19-item rating scale comprising 4 subscales (emotional/informational support, tangible, affectionate support, and positive social interactional support) of social support and one additional item. The items are rated on a 5-point rating scale ranging from “none of the time” to “to all of the time.” To obtain a score for each subscale, an average of the score for each item on the subscale is obtained. Overall support index is calculated by the average of (1) the scores for all 18 items included in 4 subscales and (2) score for the one additional item. The subscales of the MOS–SSS and the overall support index were both reported to have high reliability and Cronbach’s alpha more than 0.91. The scores have been found to be stable over time.^22^ The MOS–SSS was translated to Bengali using the same process as outlined above for the ECR-RS.

### Statistical analysis

Baseline characteristics were calculated using simple descriptive statistics. The data were checked for normality of distribution using Shapiro–Wilk test and Q-Q plots. The time interval to presentation for each patient was calculated as the dependent variable. We first ran a set of univariate regression models by regressing the time interval to presentation on a set of covariates individually. The covariates included age at interview, age when formal education was completed, urban/rural residence, partner status, current family, attachment avoidance (ECR-RS Avoidance) and anxiety (ECR-RS Anxiety) scores, and MOS scores. Additionally, we ran a multivariable model by regressing the time interval to presentation on all covariates as described earlier. For both univariate and multivariable regression analyses, we conducted generalized linear models using a gamma distribution for the errors with a log link function for model fitting. This was because the outcome variable was bounded by zero and not normally distributed.

The coefficient calculated during the univariate analysis represented the effect of a unit change of each of the independent variables on the dependent variable (i.e., logarithm of the time interval to presentation to a cancer specialist). Hence, for purposes of interpretation, we also present the exponentiated value which represents the mean ratio (MR). For example, an MR of 1.20 implies that a unit increase of the independent variable results in an increase in the mean value of time interval to presentation by 20%. Due to the log link, the difference effect on the mean of the dependent variable (the usual parameter interpretation for linear models) is not invariant to the value of independent variables. However, for the multivariable model only we also calculate the marginal difference effect averaged over the entire sample. Separate marginal plots of (1) the mean and (2) the difference in means of time interval to presentation for unit change across the full range of ECR-RS Anxiety scores were created by averaging over the sample.

## Results

We invited 155 women with OC between January 2017 and April 2018 to participate in the study. Of them, 132 (85%) women consented to participate. There were no missing data. The median age of the cohort was 50.5 (interquartile range [IQR] 42–59) years. Half (47%) of the women had a university degree and 87% lived in an urban area; 77% had a partner. Of the women, 56% lived in nuclear families with their partner and children. The remaining 44% lived within multigenerational extended families that most often included the husband’s parents and siblings. The median number of current family members living with each patient was 4 (IQR 3–6); nuclear families, 3 (IQR 2–4), and multigenerational extended families, 6 (IQR 5–7) (Table 1).

Abdominal symptoms predominated at presentation. Most patients (128/132, 97%) presented to a health professional practicing mainstream (Western) medicine. However, only 22% initially presented to a gynecologist or gynecological oncologist (Supplementary Table 1). The median time interval from onset of symptoms to presentation to an oncologist was 3 (IQR 1.25–5) months.

The majority (120/132, 90.9%) of OCs were epithelial. The most common morphological type was serous (116/132, 87.8%), followed by clear-cell,^8^ endometrioid,^6^ and mucinous.^2^ Ten women had non-epithelial OCs (Supplementary Table 1). Overall, 81% (107/132) had advanced (stage III/IV) disease. The latter (stage III/IV) was significantly associated with a time interval to presentation of more than 3 months when compared to early-stage (I/II) disease (*χ*^2^ 4.2, *P* = .03).

The median ECR-RS Anxiety score was 3.33 (IQR 2–5.25) and the median ECR-RS Avoidance score was 3.67 (IQR 3–4.33) (Table 1). There was no statistically significant association of the marital status with ECR-RS Anxiety score (Mann–Whitney U 1435, *P* = .61) and ECR-RS Avoidance score (Mann Whitney U 1241, *P* = .12). As attachment constructs are hypothesized to be stable over a lifetime, we also did not find any statistically significant association with age (ECR-RS Anxiety score *r* = −0.06, *P* = .49; ECR-RS Avoidance score *r* = −0.5, *P* = 0.6). In the present study, Cronbach’s alpha for the ECR-RS Anxiety scores was 0.79 and for the ECR-RS Avoidance scores was 0.50.

In women with a time interval to presentation of less than 3 months, the median ECR-RS Anxiety score was 3.0 (IQR 1.67–5) and median ECR-RS Avoidance score was 3.67 (IQR 3.17–4.6). For those with intervals of 3 months or more, the median ECR-RS Anxiety score was 3.33 (IQR 2–5.33) and median ECR-RS Avoidance score was 3.67 (IQR 3.0–4.33).

From both the univariate (Supplementary Table 2) and multivariate analysis (Table 2), an increased ECR-RS Anxiety score was significantly associated with longer time interval to present to a cancer specialist. There was no statistically significant association of ECR-RS Avoidance score with time interval to presentation to cancer specialist.

**Figure 1:**
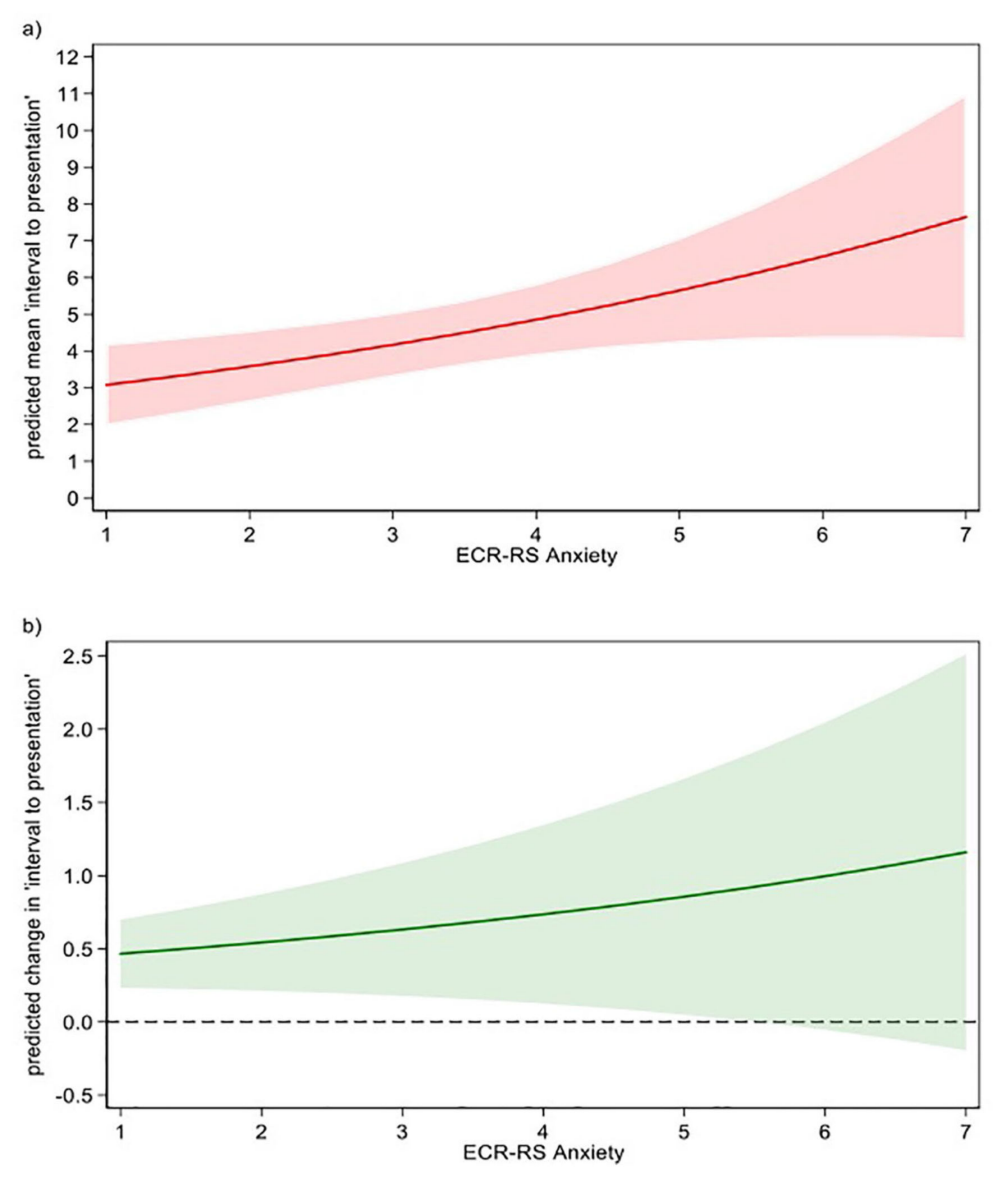
Relationship of attachment anxiety scores changes with interval to presentation (a, relationship of attachment anxiety scores changes with the mean interval to presentation; b, relationship of a unit increase in attachment anxiety score with the interval to presentation).

As the ECR-RS Anxiety scores increased from 1 to 7, the mean time interval to presentation increased from 3.1 to 7.6 months (Figure 1a), with a unit increase resulting in a 16.0% (95% confidence interval [CI]: 4%–31.1%; *P* = .019) increase in the mean time interval to presentation. The effect differed across the continuum of ECR-RS Anxiety scores. At a low score of 1, a unit increase increased the time interval to presentation by 0.5 months. However, at a high ECR-RS Anxiety score of 7, a unit increase increased the time interval to presentation by 1.1 months (Figure 1b).

The other significant factor influencing the time interval to presentation was being part of a multigenerational extended family. Compared to those living in nuclear families, these women had a 52% (95% CI: 2%–227%; *P* = .04) increase in the time interval to presentation.

Available social support as determined by MOS–SSS score was not associated with the time interval to presentation of women with OC (Supplementary Table 2; Table 2). In the present study, Cronbach’s alpha for the MOS–SSS total score was 0.91.

## Discussion

As far as we are aware, this is the first study exploring adult attachment and time interval to presentation to a cancer specialist. We found that in women with OC, increased attachment anxiety and living in a traditional multigenerational extended family were associated with delays in presentation to a cancer specialist. While the average increase in the time interval per unit increase in attachment anxiety score was 16%, the effect was more pronounced in women with high scores. There was also a 50% increase in the time interval in women from multigenerational extended compared to nuclear families.

Our findings are in keeping with the Danish study exploring time intervals and adult attachment in patients with cancer.^15^ This study, which included both male and female patients with cancer, found that adult attachment styles impacted the time interval between symptom onset and presentation to primary care physicians. In the subgroup of female patients with cancer (which included 64 with gynecological malignancies), higher attachment anxiety scores were associated with delays. That the association has been found in patients with cancer from such disparate cultural settings suggests that the finding is valid.

A number of factors may contribute to delayed presentation in patients with increased attachment anxiety. One potential explanation is the paradox that high attachment anxiety may be associated with increased rumination but longer time intervals to seeking professional help.^19^ It may lead to frequent but inconsistent requests for help, often accompanied by a poorly articulated narrative of distress. This may cause caregivers to withdraw support.^16^ In countries such as India, where there is no standardized referral pathway for cancer, patients often turn to several doctors at different hospitals for second opinions, leading to fragmented and disorganized care.^23^ This could potentially be further exaggerated in patients with attachment anxiety.

We found that being part of a traditional multigenerational extended family was associated with increased time intervals to presentation to a cancer specialist. Living in multigenerational extended families has been previously identified to be a social determinant of inequity of women’s health alongside illiteracy, financial deprivation, and poor availability of government-supported health care facilities.^24^ Previous reports from India have noted that women living in extended households are less likely to deliver babies in health care facilities or in the presence of a trained health care professional.^25^ Treatment seeking was often delayed in extended families with many women saying they had no power either to make or even influence such decisions.^26^ More “family” may not always equate to “more support.” Women who are part of large extended families have to prioritize a multitude of responsibilities and have less time to devote to their own health needs. They also often lack financial autonomy or freedom of movement.^27,28^ Other barriers to women accessing health care in India include potential loss of income,^29^ cultural inhibitions, and apprehension toward tertiary services,^30^ including a perception that health care facilities are disrespectful toward women.^31^

Our findings are contrary to a study from Botswana in Africa using univariate analysis, patients living in families with larger number of members were less likely to experience help-seeking delays for cancer care.^32^ This study comprised both men and women, with the most common cancers being advanced cervical, breast, and head and neck cancers. These cancers have a very different symptom profile to OC, where initial symptoms are nonspecific and insidious. The two populations are also quite different, with our sample consisting of mostly urban, university-educated women attending a tertiary oncology center, while 90% of the Botswana patients had only completed secondary school and were attending a free oncology service. The lack of multivariate analysis might have further contributed to this difference.

### Implications for psychosocial oncology

**Figure 2:**
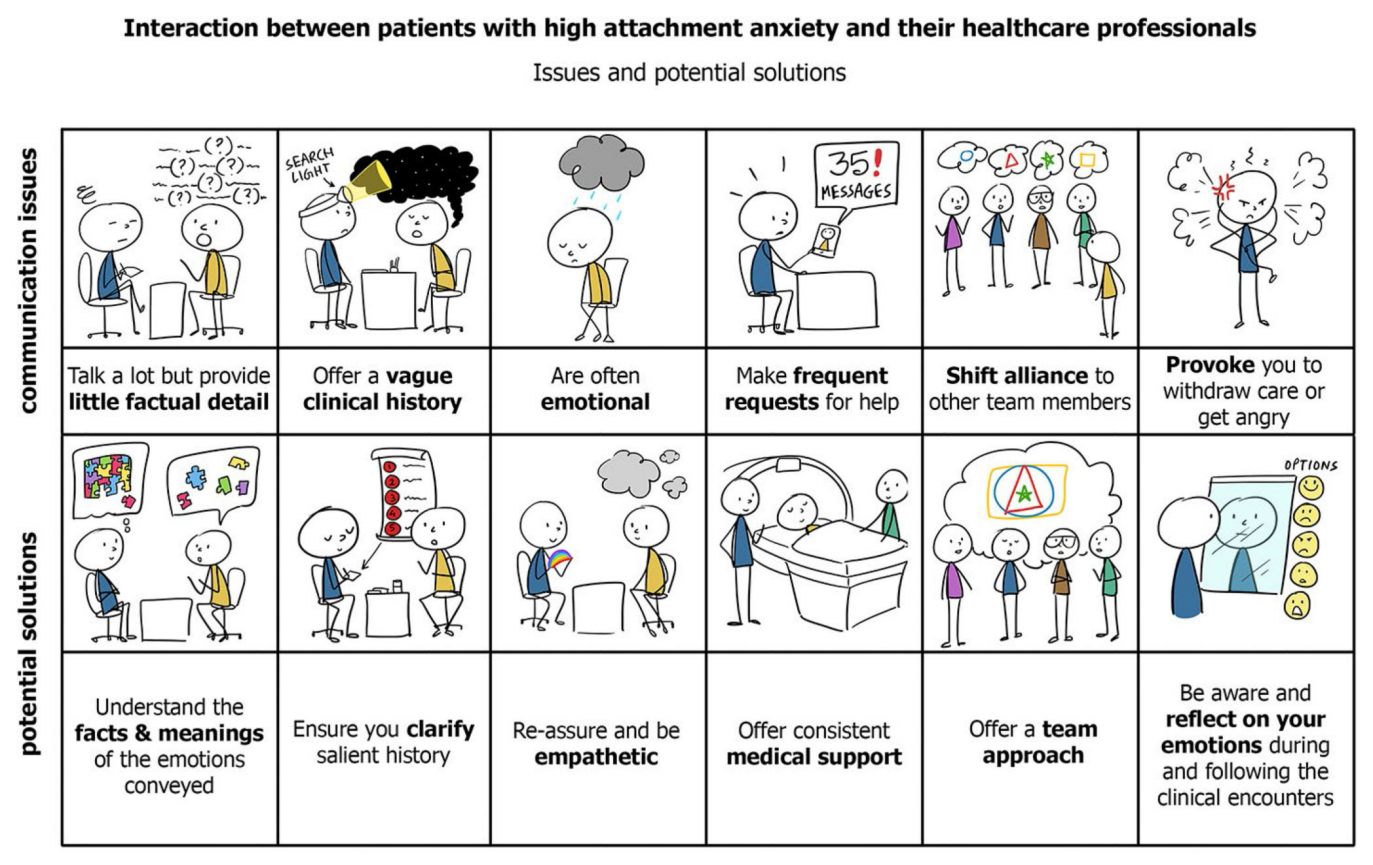
Ways to communicate with patients with high attachment anxiety.

There are currently major efforts worldwide to reduce the time to OC diagnosis. A key theme is educating primary care physicians about symptoms. If our findings are confirmed, clinicians especially in primary care also need to be made aware of insecure attachment styles and their implication for history taking and doctor–patient communication. This could help physicians make conscious efforts to continue to engage with such individuals, despite the frustration that their fragmented narratives and poorly articulated distress might provoke (Figure 2). Early recognition of insecure attachment styles could also enable appropriate referral for mental health support alongside evaluation of suspicious symptoms. Further studies are needed to explore whether the impact of adult attachment extends to help seeking in other common cancers, both in women and men.

### Study limitations/strengths

The limitations of the study include (1) the cross-sectional design, which limits inference about causality (however, it needs to be noted that attachment patterns are relatively stable over one’s lifetime); (2) recall bias affecting accuracy of patients remembering the time intervals (to minimize this, interviews were conducted soon after presentation to the cancer specialist and before start of treatment); (3) the relatively small sample size, which limited the number of sociodemographic variables we could include in the multivariate analysis; and (4) the use of overall time interval from onset of symptoms to presentation to cancer specialist, as these were two time points that patients were able to reliably report. We did not collect data on when patients first saw any health care worker and therefore cannot report on the two time intervals—patients and primary care interval^33^—that traditionally contribute to the overall time interval to diagnosis.

The strengths include (1) a homogenous sample of women with OC (the patients were educated and urban with a similar symptom profile dominated by abdominal and gastrointestinal symptoms, in keeping with most OC cohorts^1,3^); (2) a high response rate; (3) use of standardized instruments administered by trained clinical psychologists (the ECR-RS has been widely used across the world with a web-based version globally validated in a study that included participants from India^20^); and (4) adjustment for multiple confounding factors. Further, (5) we used a generalized linear model using a gamma distribution with a log link which is a natural choice for dealing with positive continuous outcomes that are typically positively skewed and have a variance that increases with mean. This model fits the data much better than a standard linear model and so provides improved statistical inference, despite the less familiar parameter interpretations. Finally, (6) the median global ECR-RS subscales scores for Anxiety and Avoidance were comparable to the mean global anxiety and avoidance scores reported in an international cohort of healthy women completing an online ECR-RS web questionnaire.^13^

## Conclusions

We found a significant independent association between attachment anxiety and delays in presenting to a cancer specialist in women with OC. There is an urgent need for this to be evaluated in independent cohorts, as it likely to have relevance to the international efforts to address delays in diagnosis of this disease. If validated, making primary physicians aware of attachment anxiety and its impact on patient assessment may facilitate earlier diagnosis and timely referral. Alongside this, there is a need to explore the role of multigenerational extended families on help seeking in OC and the impact of adult attachment in other common cancers.

## Supplementary Material

Supplementary Material

## Figures and Tables

**Table 1:** Sample characteristics and overall psychosocial variable scores.

Variables (n = 132)	Frequency (%)	Median (IQR)
Sociodemographic profile		
Age (years)		50.5 (42-59)
Domicile		
Urban	115 (87.1%)	
Rural	17 (12.9%)	
Education		
Primary school	8 (6.1%)	
Secondary school	36 (27.2%)	
Higher secondary school	22 (16.67%)	
College	36 (27.2%)	
University	26 (19.7%)	
No formal education	4 (3.1%)	
Age at which respondent left education (years)		19.5 (16–23)
Number of current family members		4 (3–5.75)
Partner status		
Single	30 (22.75)	
Has a partner	102 (77.27%)	
Type of family unit in childhood		
Nuclear	66 (50%)	
Joint	66 (50%)	
Type of family unit currently		
Nuclear	74 (56.1%)	
Joint	58 (43.9%)	
Psychosocial instruments scores		
Medical Outcome Survey		
Emotional/Informational social support subscale		4.12 (3.3–4.75)
Tangible social support subscale		4.75 (4.25–5)
Affectionate social support subscale		4.67 (4–5)
Positive social support subscale		3.67 (2.67–4.33)
Total social support index		4.01 (3.52–4.54)
Experiences in Close Relationships—Avoidance subscale score		3.67 (3–4.33)
Experiences in Close Relationships—Anxiety subscale score		3.33 (2–5.25)

**Table 2:** Multivariate model with “time to see an oncologist” as dependent variable.

	Coefficient	SE	*P* value	Mean ratio	95% mean	CI of ratio	Mean difference	Lower 95% CI	Upper 95% CI
Age	0.01	0.01	.48	1.00	0.99	1.02	0.02	−0.044	0.09
Age at which the patient stopped education	0.01	0.02	.56	1.01	0.97	1.05	0.06	−0.14	0.25
Urban/rural place of residence	−0.02	0.34	.95	0.98	0.51	1.89	−0.11	−3.16	2.95
Partner	−0.10	0.25	.69	0.90	0.55	1.48	−0.48	−2.92	1.96
Current family	0.42	0.21	.04	1.52	1.02	2.27	2.02	−0.03	4.07
Attachment related avoidance score (ECR-RS)	−0.11	0.12	.34	0.89	0.71	1.13	−0.52	−1.62	0.58
Attachment related anxiety score (ECR-RS)	0.15	0.06	.01	1.16	1.04	1.31	0.71	0.12	1.31
Social support score (MOS)	−0.01	0.16	.97	0.99	0.73	1.35	−0.02	−1.48	1.43

CI = confidence interval; ECR-RS = Experiences of Close Relationships–Relationship Style questionnaire; MOS = Medical Outcome Survey; SE = standard error.

## Data Availability

The authors declare that de-linked and anonymous data are available for the research study. Sharing of data is subject to local ethical and legal guidelines.
