# Supplementary material for: Association of adult attachment with delays in accessing specialist care in women with ovarian cancer
Source: J Psychosoc Oncol. Author manuscript; Available in PMC 2023 Jul 4. (PMC7614719; doi:10.1080/07347332.2022.2025510)
Supplement: Supplementary Material [file EMS176922-supplement-Supplementary_Material.docx]

Supplementary Table 1: Disease characteristics of the cohort

| Variable (n=132) | Frequency (%) |
| --- | --- |
| Diagnosis | |
| Epithelial | 120 (90.1%) |
| Serous | 116 (87.8%) |
| Non-epithelial | 16 (12.12%) |
| Clear cell carcinoma | 8 (6%) |
| Endometroid carcinoma | 6 (4.5%) |
| Mucinous | 2 (1.5%) |
| Stage | |
| Stage 1 | 21 (15.9%) |
| Stage 2 | 4 (3.03%) |
| Stage 3 | 61 (46.2%) |
| Stage 4 | 46 (34.8%) |
| Symptom at presentation^†^ | |
| Abdominal | 98 (74.2%) |
| GI | 44 (33.3%) |
| Gynaecological | 9 (6.8%) |
| Systemic | 46 (34.8%) |
| Others | 53 (40.1%) |
| None | 4 (3.03%) |
| First professional contact | |
| Traditional healer | 4 (3.03%) |
| General physician | 50 (37.9%) |
| Non-gynaecological specialist | 49 (37.1%) |
| Gynaecologist (non-oncologist) | 25 (20.8%) |
| Gynaecological oncologist | 4 (3.03%) |

^†^Women had multiple symptoms
